# Epidemiological considerations on HIV infection in Constanța county

**DOI:** 10.1186/1471-2334-13-S1-P20

**Published:** 2013-12-16

**Authors:** Iulia Gabriela Șerban, Sorin Rugină, Consuela Marcaş, Ghiulendan Iacub

**Affiliations:** 1 Ovidius University, Constanța, Romania; 2 HIV-AIDS Constanța Regional Center, Romania

## Background

The epidemiological trend of evolution of HIV-AIDS is a topic of great interest to the international scientific community today.

This paper aims to formulate epidemiological considerations on the evolution of HIV infection in Constanța.

## Methods

The authors performed a retrospective study on two groups of patients selected from the cohort born between 1987 and 1989, based on 2 distinct patterns of evolution: acute evolution (1987-1993) and chronic course (2009-2013).

We correlated clinical developments, therapeutic interventions and causes of death in 2 periods: 1987-1993 and 2009-2013.

## Results

Group A (patients with natural progression of the disease), with a survival rate of 18-24 months, who received only medical care and assistance, with causes of death like opportunistic infections and high mortality type.

Group B (patients with HAART), which are derived from cohort and new detected, with a mean survival time of 10-11 years, reduced mortality, predominantly through tuberculosis or pneumocystosis, polyexperimented patients, immunologically exhausted or not adherent.

## Conclusion

A retrospective epidemiological characterization of the 2 groups of patients confirmed the essential role of health care, including palliative care, especially of antiretroviral therapy in modifying the "pattern" of evolution of HIV-AIDS disease.

